# Green modification of graphene dispersion with high nanosheet content, good dispersibility, and long storage stability

**DOI:** 10.1039/d1ra08520d

**Published:** 2022-02-18

**Authors:** Minghua Li, Xiaoyu Lu, Jiajia Jiang, Lei Gao, Jie Gao, Dongming Jiang

**Affiliations:** School of Energy Materials and Chemical Engineering, Hefei University Hefei Anhui 230601 China liminghua@hfuu.edu.cn; Anhui Provincial Engineering Research Center for Green Coatings High-Performance Additives Hefei Anhui 230601 China; Hefei Aigo Additives Technology Co., Ltd Hefei Anhui 230601 China

## Abstract

In this work, an easy, green, noncovalent surface modification of pristine graphene (GR) was carried out using a single-step method between sodium carboxymethyl cellulose (CMC) and pristine GR, resulting in the formation of a modified CMC–GR (CGR) dispersion with 15% nanosheet content, the first reported in water. Results obtained from thermogravimetry analysis (TGA), Raman spectroscopy, and atomic force microscopy (AFM) confirm that the CMC modifier is successfully decorated on the pristine GR surface. Analyses of transmittance spectrum, zeta potential and transmittance electron microscopy (TEM) images reveal that the modified CGR has a high degree of dispersion. More importantly, the pristine GR is insoluble, while the modified CGR-3, mixed with 1.1 wt% CMC modifier, is easily well dispersed in water and has good flowability, and no sedimentation is observed after more than 3 months.

## Abstract 2

An easy, green, noncovalent surface modification of pristine graphene (GR) was carried out by using a single-step method, resulting in the formation of a modified CMC-GR dispersion with 15% nanosheet content, the first reported in water.

## Graphical abstract

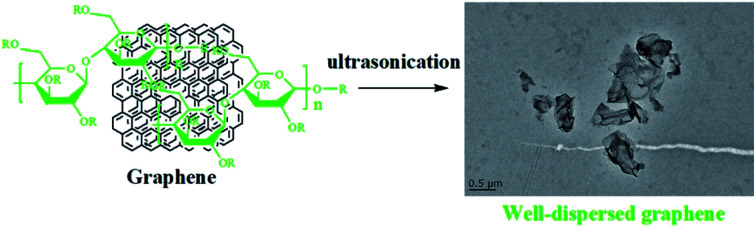

## 1. Introduction

Waterborne coatings have gained much attention for metal protection on account of their reduced emission of volatile organic compounds (VOCs), avoiding serious workplace accidents.^1^ However, waterborne coatings easily form polar channels that accelerate water permeation due to their hydrophilic groups. Thus, waterborne coatings with good corrosion resistance are urgently needed to be developed.

Polymer nanocomposites, including nanofillers such as silica,^2,3^ montmorillonite,^4,5^ boron nitride,^6–8^ and graphene,^9,10^ are considered more efficient materials that enhance the corrosion resistance of coatings by improving their barrier properties. Graphene, a promising two-dimensional (2D) nanosheet with only one-atom thickness, has a series of preeminent characteristics, such as high barrier performance, good mechanical properties, outstanding thermal stability and high chemical stability.^11–14^ At present, it has received increasing attention in the field of anticorrosion coatings for ultralight metals, and it has been used in waterborne anticorrosion coatings to enhance anticorrosion performance.^15,16^ However, graphene is not easy to disperse in water due to its high surface area, strong van der Waals forces, and intrinsic hydrophobicity. One effective method is to gain hydrophilic graphene oxide (GO) using chemical oxidation modification. Unfortunately, the defect sites introduced during the process can affect the long-term anticorrosion performance. Furthermore, many studies have been done to improve the wettability of graphene in matrix through facile noncovalent modification. Chang *et al.*^17^ constructed polyaniline/graphene waterborne epoxy coatings to enhance the corrosion resistance of steel against O_2_ and H_2_O. He *et al.*^18^ used tannic acid (TA) as intercalator to disperse graphene in water *via* π–π noncovalent bonds, forming graphene–TA hybrids and obtaining highly efficient anticorrosive epoxy coatings. Yu *et al.*^19^ prepared waterborne epoxy coatings combined with dispersed sandwiched polyvinyl butyral@graphene@polyvinyl butyral composites to improve the corrosion resistance of commercial aluminum alloys, and the effect can last 120 days even when exposed to simulated seawater.

In recent years, most works have only focused on graphene dispersion through covalent and noncovalent modification using a low graphene content, between 0.1% and 5%. However, it is difficult to use this kind of graphene dispersion as a corrosion resistance additive for industrial waterborne coatings. Based on the demand for corrosion resistance and mass production requirements, graphene dispersions with high solid content and good dispersibility in water need to be designed and developed.

**Scheme 1:**
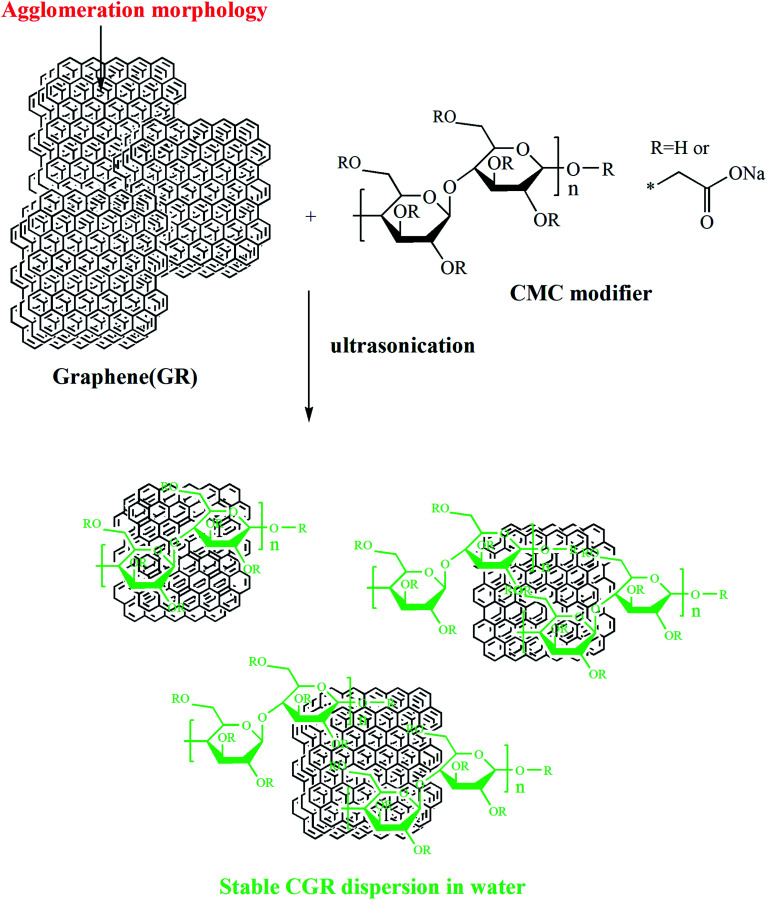
The preparation route for sodium carboxymethyl cellulose (CMC)-modified CGR dispersion.

In this paper, we report an easy, green modification of graphene by using a noncovalent strategy between CMC and graphene, as shown in Scheme 1, to prepare a modified graphene CGR dispersion with 15% nanosheet content, good dispersibility, and long storage stability, the first reported in water.

This method for green surface modification of pristine GR can be used to prepare GR/waterborne polymer nanocomposites. The entanglements between the long chains of CMC and the polymer matrix substantially enhance the interactions between them and accordingly improve their compatibility. Importantly, this strategy is available and economical due to its easy operation in industry. We also believe that this work will accelerate the development of industrial anticorrosion coatings for ultralight metals.

## 2. Experimental

### 2.1 Materials

Pristine graphene (GR) nanosheets (black powder, D50: <10 μm, BET: 180–280 m^2^ g^−1^) were obtained from Sixth Element (Changzhou) Materials Technology Co. Ltd Sodium carboxymethyl cellulose (CMC, anionic surfactant) was purchased from Hefei Aigo Additives Technology Co. Ltd. Deionized water was prepared using the appropriate equipment.

### 2.2 Modification of GR with CMC modifier

First, deionized water ((85–*n*) g) and CMC modifier (*n* g) water were added to a three-necked flask and stirred with a high-speed stirrer at 900 rpm for 15 minutes. Then, crude GR (15 g) was added to the preprepared mixture and sonicated for 1 hour to obtain a uniform CGR dispersion. For the sake of comparing different CGR dispersions, four different contents of CMC modifier (*n* = 0.7, 0.9, 1.1 and 1.3 wt%) were designed to prepared CGR-*x* (*x* = 1, 2, 3 and 4), correspondingly. Then, the products were purified after each modification by ultrafiltration. The resulting CGR solid powders were dried overnight at 80 °C in vacuum.

### 2.3 Characterization

Thermogravimetry analysis (TGA) of pristine GR and CGR was performed under nitrogen on a STA409PC thermogravimetric analyzer (Netzsch instruments) with a temperature range of 25–1000 °C at a heating rate of 10 °C min^−1^. Raman spectroscopy of pristine GR and CGR was carried out using a Kaiser Holo-Lab 5000 series spectrometer furnished with a 514 nm excitation laser. The particle size distribution and zeta potential of the graphene dispersion were characterized by a 90Plus PALS Zeta Potential (Brookhaven Instruments, USA). The transmittance of the graphene dispersion was investigated with an ultraviolet spectrophotometer (UV2600). Morphology of the graphene dispersion was investigated by field emission scanning electron microscopy (FE-SEM, SU8010) and transmittance electron microscopy (TEM, JEOL JEM2011). Atomic force microscopy (AFM, Park XE7) was used to characterize the thickness of the GR nanosheets.

## 3. Results and discussion

### 3.1 TGA measurements

**Fig. 1:**
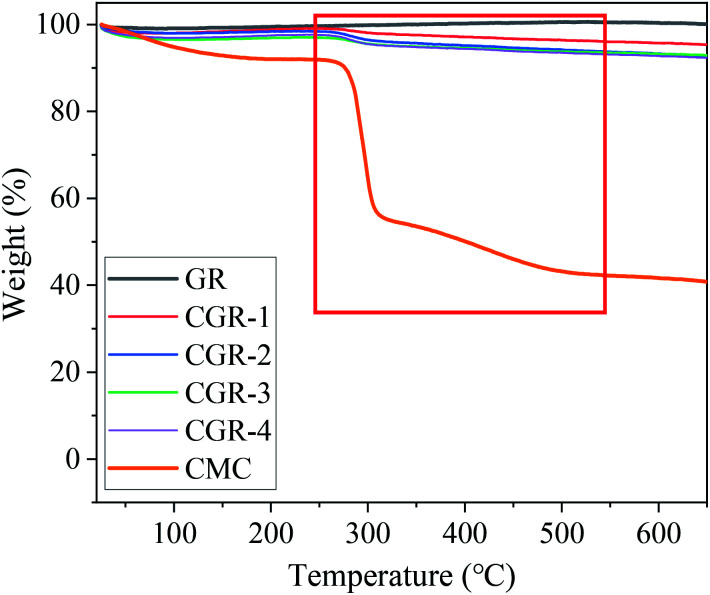
TGA curves of pristine GR, modified CGR, and CMC modifier.

Supporting evidence for the noncovalent attachment of CMC modifier on the pristine GR surfaces comes from the thermogravimetry analysis (TGA). Fig. 1 shows TGA curves for the analysis of pristine GR and modified CGR with different contents of CMC at a temperature ramp rate of 10 °C min^−1^, respectively. The first stage of mass loss terminates at approximately 180 °C, which is due to the rejection of the adsorbed water from the interlayers of the materials. The TGA plot of CMC indicates a gradual mass loss of around 58.4% as the temperature reached 600 °C. Furthermore, we noted that CGR showed a significant weight loss in the range of 260–600 °C, corresponding to pyrolysis of the CMC modifier. The results indicate that the CMC modifier can be adsorbed on the surface of graphene.

### 3.2 Raman spectroscopy

**Fig. 2:**
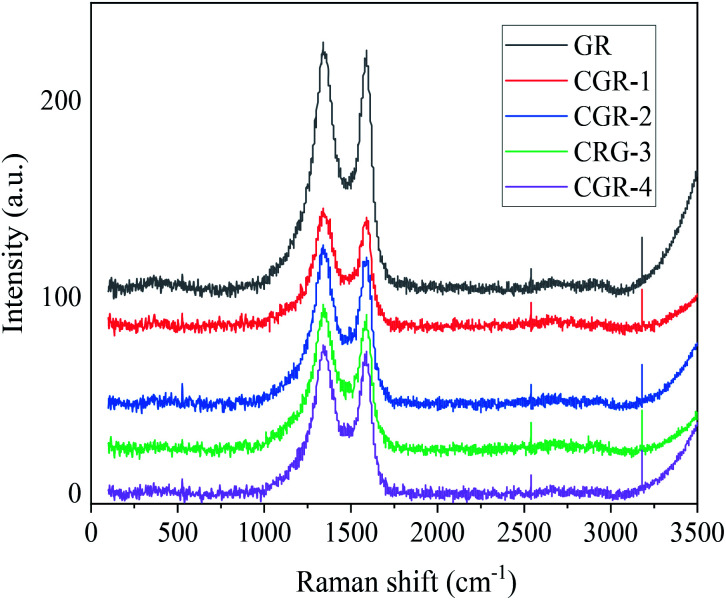
Raman spectroscopy of pristine GR and modified CGR.

Raman spectroscopy is usually employed to distinguish the ordered and disordered carbon structures of graphene.^18^ The D band and G band are represented in the in-plane vibration of sp^2^ carbon atoms and the vibration of sp^3^ carbon atoms from the functional groups, respectively. It can be seen from Fig. 2 that pristine GR exhibited two characteristic peaks at 1342 cm^−1^ (D band) and 1586 cm^−1^ (G band), *I*_D_/*I*_G_ = 1.03. The *I*_D_/*I*_G_ ratio presents a slight increase from 1.03 for pristine GR to 1.06 for CGR-4, indicating that no more defects are introduced after the modification of pristine GR with CMC modifier, and the graphene preserves its basic structural properties.

### 3.3 Size and distribution

**Fig. 3:**
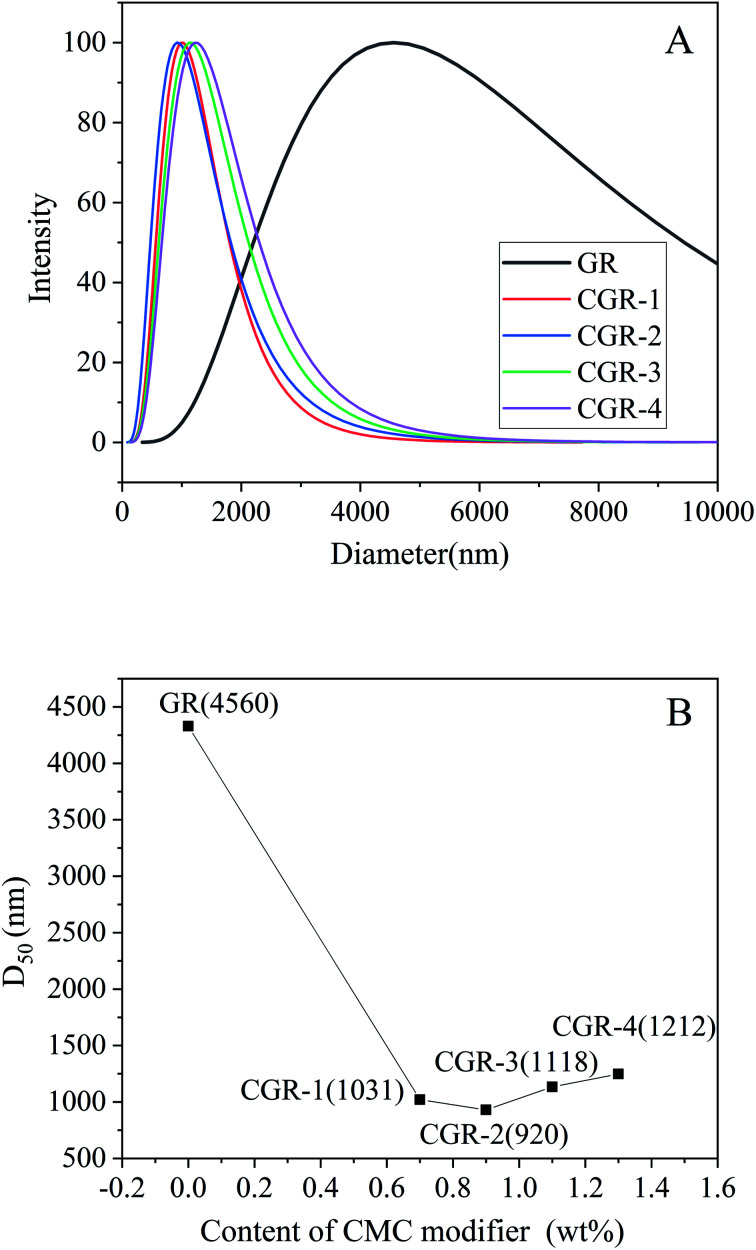
(A) Size and distribution of pristine GR and modified CGR dispersions. (B) Relation between D_50_ and content of CMC modifier in the GR nanosheets.

The diluted dispersions of pristine GR and CGR with obvious Tyndall effect in water were prepared through mechanical dispersion for 15 min. On the basis of the dynamic light scattering (DLS) principle, the average diameter and size distribution of the graphene dispersions were analyzed using a zeta potential analyzer, as shown in Fig. 3.

According to the result, the pristine GR was dispersed poorly in water, and serious agglomeration was formed because of the high surface area, strong van der Waals forces, and intrinsic hydrophobicity of GR. Its average diameter (D_50_) is about 4560 nm, as shown in Fig. 3B. Compared with pristine GR, the CGR modified with CMC showed good dispersibility in water, especially CGR-2 (920 nm), which is mixed with 0.9 wt% modifier. It was suggested that the dispersion effect of graphene using ultrasonic exfoliation in water can be improved after modification with a macromolecular modifier. Because of the new noncovalent modification between CMC and GR nanosheets, the agglomeration was controlled effectively. With the increasing content of CMC modifier, the average diameter of graphene decreased gradually, but after the content of CMC modifier exceed 0.9 wt%, the average diameter increased again. This may be because when the content of CMC is insufficient, the uncoated graphene will be likely to agglomerate, while if the CMC content is superabundant, the average diameter of the modified CGR may increase again because of thickening of the CMC coating layer and entanglement among the long and flexible chains of the macromolecular modifier.

### 3.4 Transmittance spectrum

**Fig. 4:**
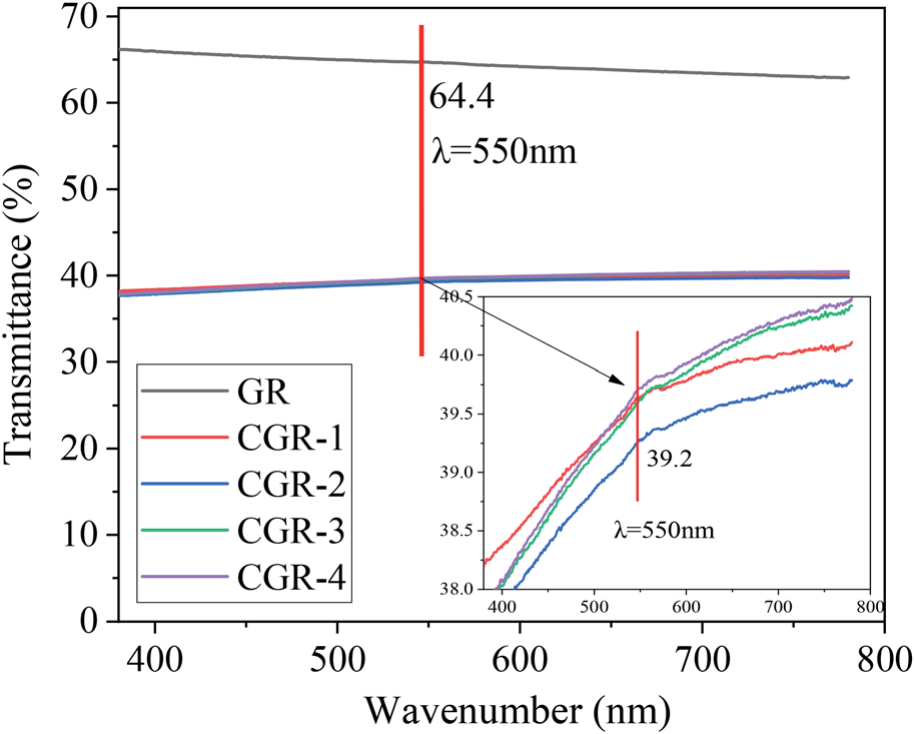
Transmittance spectra of the dispersions of pristine and modified CGR samples with incident light wavelength ranging from 400 nm to 800 nm. The data in the figure indicate the optical transmittance with incident 550 nm visible light.

For quickly evaluating the dispersion effect of graphene, it is necessary to compare the optical transmittance. Fig. 4 shows the transmittance spectra of dispersions of pristine GR and modified CGR, with incident light wavelength ranging from 400 nm to 800 nm. The number in the figure indicates the optical transmittance at 550 nm incident light. We extracted the experimental data at 550 nm from pristine and modified CGR in Fig. 4. As seen from the enlarged inset, we can conclude that the four modified CGR dispersions have similar optical transmittance values, at around 39.2%. Compared to 64.4% at 550 nm for pristine GR dispersion, the optical transmittance of the modified CGR is lower, indicating that the dispersibility of GR has been improved by the CMC modifier.

### 3.5 Dispersibility and stability

**Fig. 5:**
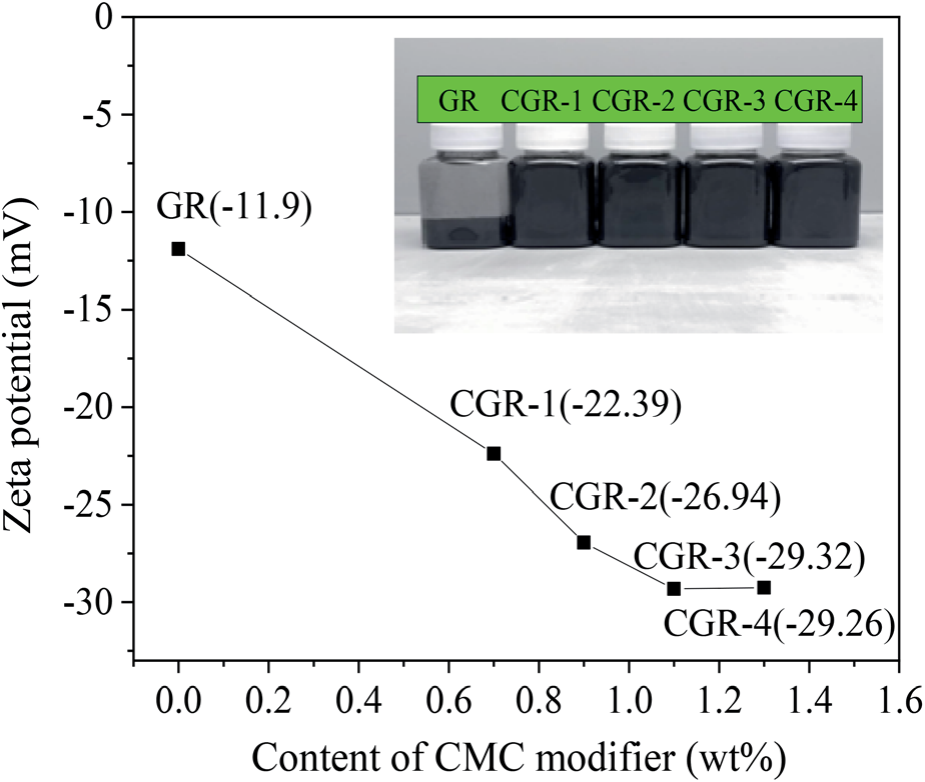
Relation between zeta potential and content of CMC modifier for the GR nanosheets. The inset photo shows the dispersibility of pristine GR and modified CGR in water.

The most important parameter defining surface properties of electrostatically stabilized nanomaterials in aqueous solutions is the zeta potential value. The relation between zeta potential and CMC modifier content in the GR nanosheets is shown in Fig. 5. This plot shows that the zeta potential decreases with increasing CMC until the content of CMC modifier reached 1.1 wt%. The plot seems to keep balance when the content of CMC modifier was increased; then, the zeta potential reaches the maximal absolute value at 29.3 mV. Therefore, it can be presumed that there are more carboxylate groups of the CMC modifier on the surface of the GR nanosheets when the absolute value of the zeta potential is high. In other words, the stability of GR dispersions has been improved due to high zeta potential.

The inset photo in Fig. 5 shows the dispersibility of pristine GR and modified CGR in water. The dispersibility of the CMC-modified GR is much better than that of their physical mixture. The sample of physical mixture has obvious black sediments in water. In contrast to CGR modified with different contents of CMC coated on the surface of graphene, the pristine GR is insoluble, while the CGR-3 dispersion mixed with 1.1 wt% CMC modifier was easily well dispersed in water, and no sedimentation was observed after more than 3 months. This suggests that CGR-3 has excellent solubility in water. However, 0.7–0.9 wt% CMC-modified GR dispersions had a little sediment, indicating that the content of CMC modifier was not enough to bring mutual exclusion and steric hindrance effects. When the content of CMC modifier is 1.3 wt%, the GR dispersion has bad flowability due to the higher viscosity. The above results demonstrate that the CGR dispersion is a physically stable system.

### 3.6 Surface morphology

**Fig. 6:**
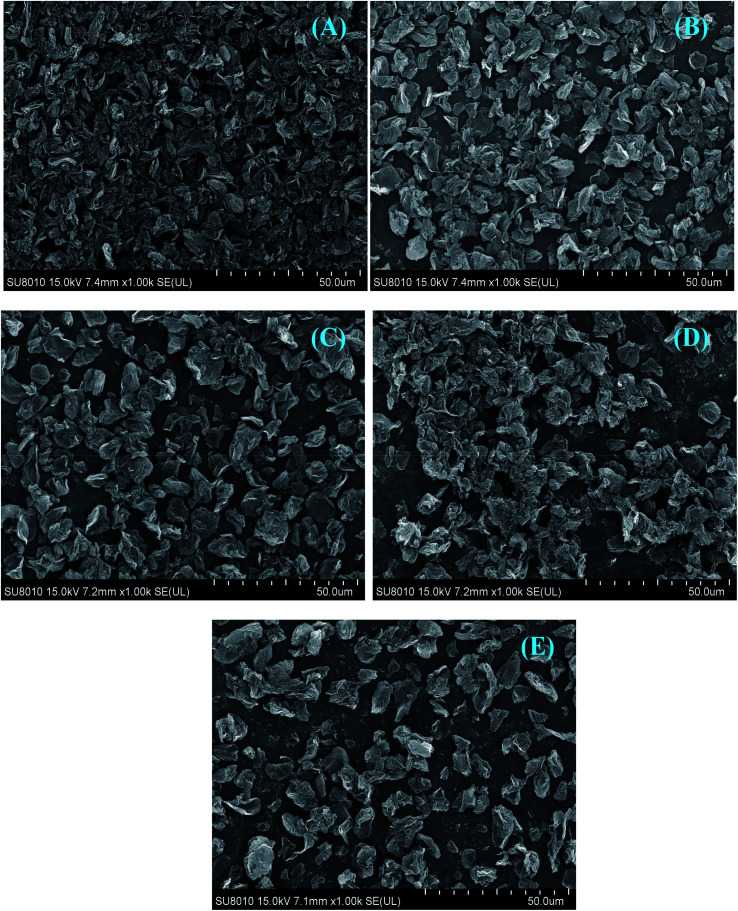
(A–E) SEM images of pristine GR (A), CGR-1 (B), CGR-2 (C), CGR-3 (D), and CGR-4 (E) drop-casted from the water solutions.

The surface morphologies of pristine GR and modified GR dispersed in water were examined by SEM, as shown in Fig. 6. The pristine GR nanosheets dispersed in water at the Si substrate show tight agglomeration,^18,20^ which is attributed to the poor dispersion of GR nanosheets (Fig. 6A). In comparison, the aggregation of modified CGR is reduced, which is due to the modification of CMC.

**Fig. 7:**
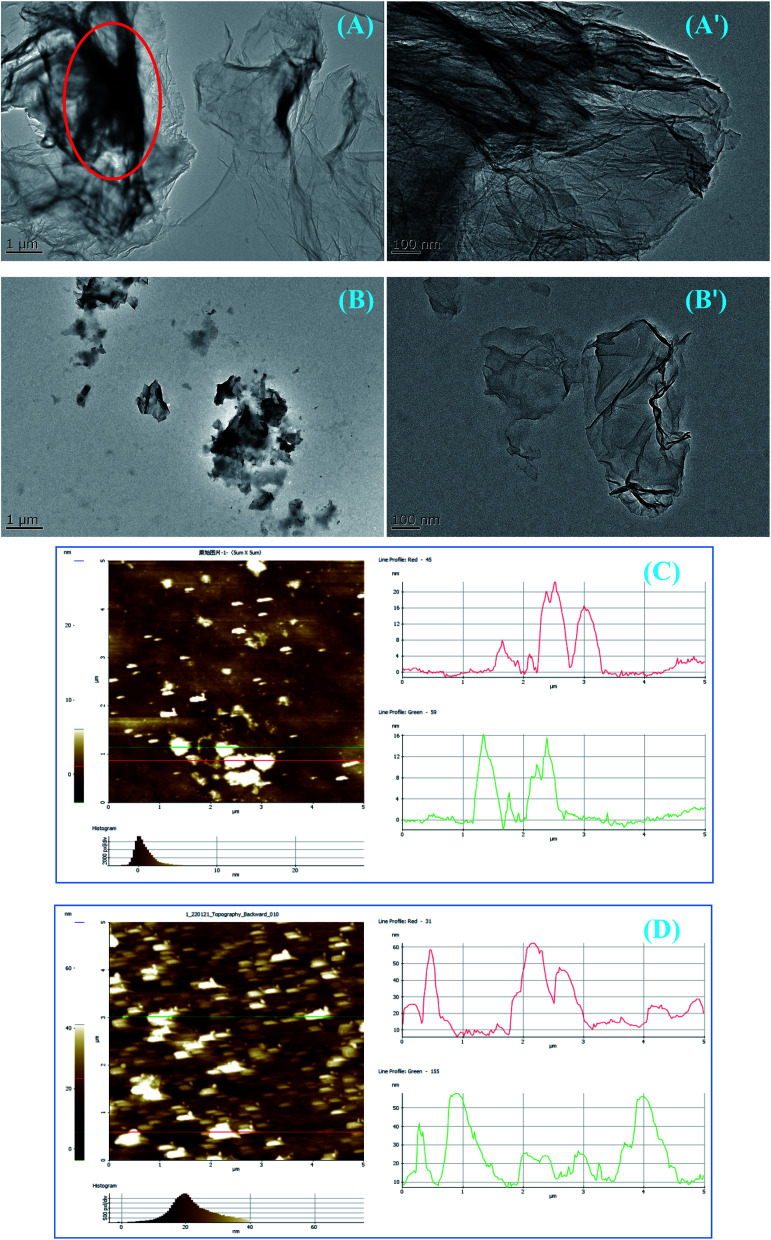
TEM and AFM images of (A and C) pristine GR and (B and D) modified CGR-3 drop-casted from the water solutions, showing different dispersion morphologies.

Furthermore, TEM results confirmed the dispersibility of pristine GR and modified CGR-3 with the best dispersion stability in Fig. 7. The pristine GR nanosheets dispersed in water at the Cu grid also show tight agglomeration and exist as large sheets, which is due to the poor dispersion (marked as red circles in Fig. 7A) of GR nanosheets. After modification with CMC, the CGR-3 nanosheets are well dispersed in water, show good dispersibility, and exist as small nanosheets (Fig. 7B), indicating that CGR dispersion is easy to disperse in waterborne coatings and builds good barrier properties. The thickness of pristine GR and modified CGR-3 was further examined by AFM. As shown in Fig. 7C, the thickness of the GR layers reaches about 16–20 nm. The thickness of CGR-3 nanosheets with the thick layer is 40–60 nm, indicating that the increase in thickness is due to the presence of CMC modifier on the GR nanosheets (Fig. 7D). Thus, it can be inferred from the increased thickness that the GR surface was successfully modified with CMC.

## 4. Conclusion

In summary, a CMC-modified CGR dispersion was successfully prepared in aqueous solution by single-step noncovalent functionalization technique. The results show that the optimum content of CMC modifier coated on the surface of graphene is 1.1 wt%. The modified CGR possesses good dispersibility and good flowability in water. The prepared CGR-3 dispersion, with no sedimentation after more than 3 months, has potential application as a functional corrosion resistance additive for waterborne coatings, providing a new strategy for the high-value-added use of graphene.

## Conflicts of interest

There are no conflicts to declare.

## Supplementary Material
